# Immunotherapy, targeted therapy, and their cross talks in hepatocellular carcinoma

**DOI:** 10.3389/fimmu.2023.1285370

**Published:** 2023-12-19

**Authors:** Jun Liu, Kevin Park, Ziyang Shen, Hannah Lee, Purnima Geetha, Mohammadreza Pakyari, Li Chai

**Affiliations:** ^1^ Department of Pathology, Brigham and Women’s Hospital, Boston, MA, United States; ^2^ Case Western Reserve University School of Medicine, Cleveland, OH, United States; ^3^ University of California, San Diego, CA, United States; ^4^ Northeastern University, Boston, MA, United States

**Keywords:** hepatocellular carcinoma, biomarker, precision medicine, transcription factors (TF), combination therapy, immunotherapy

## Abstract

Hepatocellular carcinoma (HCC) is a challenging malignancy with limited treatment options beyond surgery and chemotherapy. Recent advancements in targeted therapies and immunotherapy, including PD-1 and PD-L1 monoclonal antibodies, have shown promise, but their efficacy has not met expectations. Biomarker testing and personalized medicine based on genetic mutations and other biomarkers represent the future direction for HCC treatment. To address these challenges and opportunities, this comprehensive review discusses the progress made in targeted therapies and immunotherapies for HCC, focusing on dissecting the rationales, opportunities, and challenges for combining these modalities. The liver’s unique physiology and the presence of fibrosis in many HCC patients pose additional challenges to drug delivery and efficacy. Ongoing efforts in biomarker development and combination therapy design, especially in the context of immunotherapies, hold promise for improving outcomes in advanced HCC. Through exploring the advancements in biomarkers and targeted therapies, this review provides insights into the challenges and opportunities in the field and proposes strategies for rational combination therapy design.

## 1 Introduction

Hepatocellular carcinoma (HCC) is the most prevalent form of primary liver cancer, accounting for 75-90% of all cases. It is characterized by a high mortality rate and ranks as the third leading cause of cancer-related deaths worldwide. It is responsible for over 600,000 deaths annually with the highest incidence in Asia, and its incidence and mortality in Europe and the United States are rising steadily. Effective treatments are still lacking, and the 5-year overall survival rate remains extremely poor. The prognosis of HCC heavily depends on the stage at the time of diagnosis. While surgery, liver transplantation, or radiological intervention may be viable options for early-stage disease, especially under a multidisciplinary tumor board (MDT) setting (1, 2), the prognosis for advanced-stage HCC remains bleak, with most patients succumbing within 20 months after diagnosis. Unfortunately, even with available treatments for early-stage patients, the majority of HCC cases still progress to advanced stages, presenting a significant clinical challenge (3, 4). For many years, combination chemotherapy has not been shown to improve overall survival but has nonetheless been in wide usage due to its possible benefit in palliation.

HCC is associated with various risk factors, with the most common being hepatitis B virus (HBV) or hepatitis C virus (HCV) infection, long-term alcohol consumption, and non-alcoholic fatty liver disease (NAFLD). Other less common causes include obesity, diabetes, aflatoxin exposure, hereditary hemochromatosis, smoking, tyrosinemia, and glycogen storage disease type 1a (5, 6). The pathophysiology of HCC is complex, and hepatocarcinogenesis is not yet fully understood. It is believed that the accumulation of genetic and epigenetic changes during cirrhosis contributes to the development of HCC by deregulating the cell cycle and suppressing apoptosis. Telomere dysfunction and alterations in the micro and macro environment have been implicated as possible mechanisms accelerating HCC development in cirrhotic livers (7, 8). The diverse etiologies lead to various HCC subtypes that may respond preferentially to therapies (9).

Transcription factors (TF) such as Spalt-like protein 4 (SALL4) and signaling pathways such as Ras/Raf/Mitogen-activated protein kinase/ERK kinase (MEK)/extracellular-signal-regulated kinase (ERK) pathway, phosphatidylinositol-3-kinase (PI3K)/Akt/mechanistic target of rapamycin (mTOR) pathway, Wnt/β-catenin pathway, and Janus kinase/signal transducer and activator of transcription (JAK/STAT) pathway have been identified as key players in HCC (7). These pathways are directly regulated by receptor tyrosine kinases (RTK). A burgeoning number of targeted therapies have been developed with many of them targeting RTKs such as epidermal growth factor receptor (EGFR), vascular endothelial growth factor receptors (VEGFR), and platelet-derived growth factor receptor (PDGFR).

In addition to RTKs, immune checkpoints are key players in regulating HCC. Immune checkpoints play a vital role in moderating immune responses to prevent autoimmune diseases. HCC tumors can upregulate the expression of Programmed Death Ligand 1 (PD-L1), which then interacts with Programmed Death Protein 1 (PD-1) on T cells, effectively suppressing the immune response. This interaction helps the tumor cells evade immune surveillance and clearance, leading to tumor progression. Immune checkpoint inhibitors (ICI) are now widely used in multiple tumor types including HCC.

However, it is crucial to note that these current “gold standard” treatments for advanced HCC primarily aim to extend the lives of patients rather than to provide a cure. Sorafenib is a multikinase inhibitor (MKI) designed to target RTKs such as VEGFRs and PDGFR, but it suffers from a low response rate of 1-2%. A large clinical trial involving centers in Europe and America (SHARP trial) showed that it improved median overall survival of around 2 months (10). A following trial in Asia Pacific (Sorafenib AP trial) showed similar results, improving median overall survival from 4.2 months to 6.5 months (11). Other kinase inhibitors developed to target HCC include Regorafenib by Bayer (12) and Lenvatinib by Merck (13) showed improvement of median survival around 3 months.

These less than satisfactory results showed an urgent need to look for disruptive therapeutic options for the benefit of HCC patients. In recent years, there have been remarkable transformations in the standard of care (SOC) and the available options for HCC treatment in the advanced stages, where the unmet needs are the highest. The recent approval of ICI + VEGF combination therapy, Avastin + Tecentriq (atezolizumab plus bevacizumab), by the Food and Drug Administration (FDA) in May 2020 led to its replacement of the SOC in first-line advanced HCC, shifting MKIs increasingly towards second-line options. Although the IMBrave150 trial of Tecentriq-Avastin initially demonstrated a 42% reduction in the risk of death and a 41% reduction in the risk of disease progression or death compared to Sorafenib, an updated analysis performed 12 months after the primary analysis of IMbrave150 showed that Median OS was only 5.8 months longer with atezolizumab plus bevacizumab than sorafenib (14, 15). Nonetheless, its relative success channeled tremendous enthusiasm and optimism towards the use of immunotherapy and combination approaches, such as combining ICI and targeted therapies, in a new era of HCC drug development.

In addition to advanced HCC, there is ongoing development and potential approval of systemic therapies in combination with transarterial chemoembolization (TACE) for intermediate-stage patients and in the adjuvant setting (16). Adjuvant treatments may also hold promise for reducing recurrence rates in early-stage patients. In line with general trends in oncological drug development, systemic therapy is expected to be more effective and less associated with adverse effects, especially for intermediate-stage patients who already have access to locoregional therapy (17). These trends call for combining different mechanisms of action (MOAs) and expanding into precision-targeted therapy.

In this comprehensive review, we aim to delve into the advancements in biomarkers and targeted therapies for HCC, exploring the challenges, opportunities, and recent developments in the field. We will discuss the different mechanisms of action of pathogenic pathways that have led to the design of recent and emerging targeted therapies and combination therapies. We will also discuss the ongoing effort of biomarker development, and we will propose ways of combination therapy can be rationally designed, especially in the setting of immunotherapy which has gained the strongest traction in the recent years for the treatment of advanced HCC.

## 2 HCC biomarkers: from disease surveillance to therapeutic targeting

### 2.1 HCC biomarkers for screening and diagnosis: current standard of care

**Figure 1:**
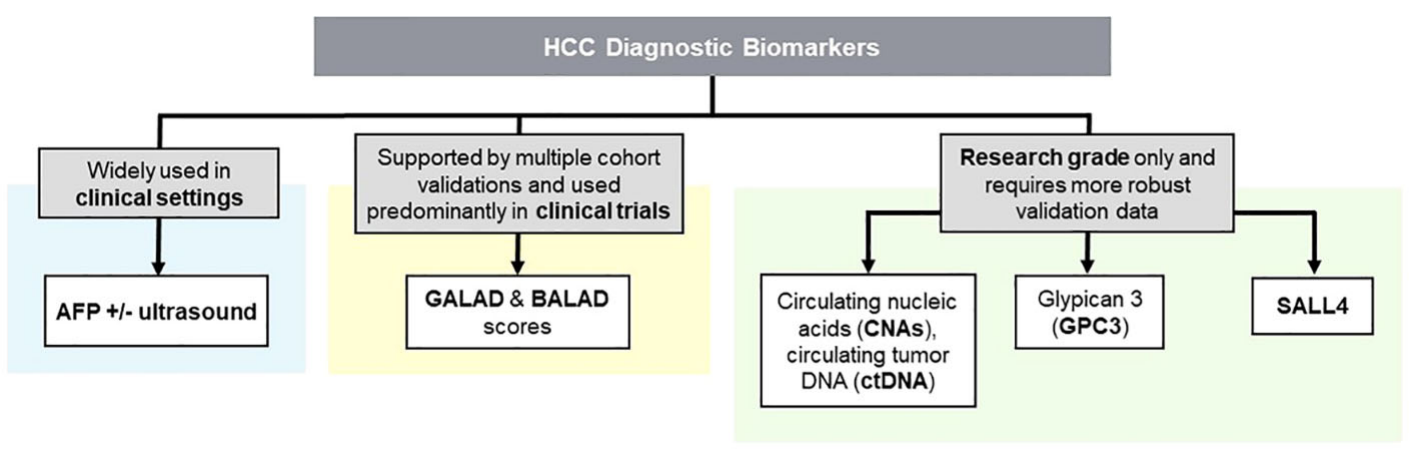
Clinical and Research Grade HCC Diagnostic Biomarkers; an Overview. Biomarkers in HCC are classified into clinical and research categories. While clinical biomarkers aid accurate patient assessment such as GALAD and BALAD scores, ongoing research aims to develop novel biomarkers for improved diagnosis and treatment.

Early detection and diagnosis of HCC is crucial for effective treatment and improved patient outcomes. As an important piece of the puzzle for precision medicine, diagnostic biomarkers are not only crucial for accurate diagnostic evaluation, risk stratification, and prognosis, but are also playing increasingly important roles in assisting/guiding targeted therapies. Currently, The most commonly used surveillance modalities include ultrasound, serum alpha-fetoprotein (AFP) measurement, or a combination of both. AFP is an “oncofetal” protein produced by the liver and yolk sac during fetal development. It is re-expressed by HCC cells and its upregulation can be readily detected via routine blood tests (18–20). Although it is the most widely used serum biomarker for HCC surveillance and monitoring, it has suboptimal specificity due to higher AFP expression in non-cancerous liver regeneration (21). According to the American Association for the Study of Liver Diseases (AASLD) Practice Guidance, it recommends HCC surveillance using a combination of liver ultrasound and AFP with a sensitivity in detecting early-stage HCC of 63% (95% CI, 48%-75%) (**Figure 1**). HCC diagnosis can be made in at-risk patients based only on specific noninvasive imaging criteria without histologic confirmation, via contrast-enhanced multiphase computed tomography CT or magnetic resonance imaging (MRI) (22).

### 2.2 Composite HCC biomarker scores under development

A combination of multiple biomarkers may improve the accuracy and sensitivity of HCC screening, as demonstrated by the development of GALAD and BALAD scores, both of which have been extensively validated in cohort studies. (**Figure 1**) The GALAD score is an example of a combination diagnostic method incorporating AFP, patient’s age, gender, and two other biomarkers: des-carboxy-prothrombin (DCP) and lens culinaris agglutinin-reactive fraction of AFP (AFP-L3) (23). In a cohort of Caucasian patients with chronic viral hepatitis, the GALAD model demonstrated an area under the receiver operating characteristic curve (AUC) of 0.94 when differentiating HCC from non-HCC. This value was found to be higher than AUCs of 0.86, 0.83, and 0.83 when AFP, AFP-L3, and DCP biomarkers are used alone, respectively (24). Furthermore, a meta-analysis of 15 studies with 19 cohorts from both Western and Eastern countries yielded a sensitivity, specificity, and AUC of 0.82, 0.89, and 0.92, with approximately 70% chance of detecting early-stage tumors (23). Similarly, the BALAD score is a surveillance method using 5 serum markers: bilirubin, albumin, AFP-L3, AFP, and DCP. In a study involving 2,600 HCC patients across 5 Japanese institutions, BALAD demonstrated a comparable ability to predict patient survival to both tumor stages and other scoring systems. These include the Cancer of the Liver Italian Program and Japan Integrated Staging scoring systems, which account for tumor progression and remnant liver function (25). In a North American cohort consisting of 148 patients, the BALAD score was found to be significantly correlated to patient survival, with its performance comparable to Barcelona Clinic Liver Cancer staging and GALAD scores (26). In addition to BALAD and GALAD scores, other AFP-based multi-biomarker algorithms have been attempted in the research setting such as the HCC Early Detection Screening (HES) algorithm which incorporates current AFP and change in AFP over the last year, age, platelets, alanine aminotransferase (ALT) for HCC screening (27). A study on a cohort of patients with liver cirrhosis from the University of Michigan utilized a prospective-specimen-collection, retrospective-blinded-evaluation design to assess the accuracy of the HES algorithm. It exhibited an AUC of 0.70 when screening for early stage HCC, and a sensitivity of 34.6% to 45.2% and specificity of 90.5% for any-stage and early stage HCC.

### 2.3 Emerging novel HCC biomarkers

Recent studies have explored novel serum/plasma biomarkers, such as circulating nucleic acids (CNAs), circulating tumor DNA (ctDNA), Glypican-3 (GPC3), and SALL4 (**Figure 1**). CNAs are fragments of DNA or RNA that are released from cancer cells into the bloodstream and can be detected using non-invasive liquid biopsy techniques (28). Several studies have reported that CNAs, such as circulating tumor DNA (ctDNA) and microRNAs (miRNAs), can serve as promising biomarkers for HCC diagnosis, prognosis, and treatment response monitoring (29–31). ctDNA refers to small fragments of DNA that are released into the bloodstream by tumor cells. Several studies have demonstrated the potential of ctDNA as a diagnostic biomarker for HCC. For example, a study by Zhao et al. (2020) found that ctDNA could accurately detect HCC with a high sensitivity and specificity (32). It may outperform conventional biomarkers such as AFP for the early detection of HCC, especially in patients with small tumors or those at high risk for HCC development.

GPC3 and SALL4 are two other oncofetal protein biomarkers with similar properties to AFP, and their roles in HCC screening/diagnosis are an area of active investigation. GPC3 is a proteoglycan anchored to the cell membrane that is normally detected in the fetal liver but not in the healthy adult liver. However, GPC3 is overexpressed at both the RNA and protein levels in HCC patients, making it a promising HCC biomarker candidate. Serum levels of GPC3 can be detected using enzyme-linked immunosorbent assay (ELISA), and immunohistochemistry (IHC) can confirm its expression in tumor tissues (33). GPC3 expression has been observed in malignant hepatocytes in hepatoblastomas and in 84% of HCC (33). In addition, GPC3, in combination with AFP and/or imaging techniques such as ultrasound, CT, and MRI, has shown high sensitivity and specificity for the diagnosis of HCC (34). While GPC3 alone has a sensitivity of 55.1% and specificity of 97%, a combination of GPC3 and AFP has a sensitivity of 75.7% and specificity of 83.3% for HCC diagnosis when tumors are less than 3 cm (35). In addition to its use in diagnosis, GPC3 is also being studied as a potential prognostic biomarker for HCC. Studies have shown that GPC3 expression levels in HCC tissues are associated with tumor aggressiveness and poor prognosis, including tumor size, vascular invasion, and metastasis (36, 37). Elevated serum levels of GPC3 have also been associated with worse overall survival and disease-free survival in patients with HCC (38). Based on these data, GPC3 may be useful for stratifying HCC patients based on their prognosis and guiding treatment decisions.

SALL4 is a zinc finger TF involved during fetal liver development that acts as a HCC oncogene and is highly expressed in HCC cells (39–41). In contrast to GPC3 and AFP, which can be highly expressed in regenerating liver tissue affected by non-neoplastic lesions such as hepatitis (GPC3 and AFP) and liver cirrhosis (AFP) (21, 33), SALL4 serological levels were found to be considerably higher in HCC patients compared to patients with cirrhosis or chronic hepatitis, potentially offering better specificity as a biomarker. A similar trend was observed in SALL4 expression levels across HCC tissues, as compared to liver hemangioma and adjacent noncancerous hepatic tissue (39, 41). SALL4 expression is correlated to poor prognosis and serves as a prominent biomarker predicting HCC progression (39–41). Interestingly, there has been a wide variation in SALL4 positivity observed from different publications focusing on different patient demographics. Analysis of SALL4 expression in Eastern regions such as China and Singapore demonstrated a significantly upregulated SALL4 expression in HCC tissues relative to non-neoplastic tissues. Approximately 46% and 55.6% of HCC tissue specimens from the Chinese (n = 126) and Singaporean (n = 171) cohorts exhibited SALL4 positivity (40, 41). In studies concerning Western regions, 26% and 46% of HCC tumors from patients in the Netherlands (n = 133) and United States (n = 69) cohorts were positive for SALL4 (42, 43). Contrastingly, an analysis of 236 cases in Washington state yielded only 3 cases positive for SALL4 (1.3%); however, the validity of the results remains questionable due to multiple shortcomings of the study which include a deviation from published liver carcinoma staining protocols and the use of a core biopsy method known to be highly susceptible to sampling bias and false negatives (44). In Eastern regions, HBV infections are the most common causes of HCC cases, with HBV-positive patients composing 31 to 91% of total HCC patients across multiple studies (45–50). In Western regions, the prevalence is relatively lower, with approximately 12 to 23% of HCC patients demonstrating HBV-positivity (42, 51). Given the upregulated SALL4 expression in nearly 50% of HBV-related HCCs, it is speculated that HBV infection may be associated with SALL4 re-expression (40). In a study by Fan et al, HBV-related HCCs exhibited significant hypomethylation in the regulatory region of SALL4. The same DNA demethylation patterns were observed in liver cancer cell lines and HBV- and HCV related HCCs with SALL4 overexpression attributable to increased STAT3 and OCT4 binding at the respective hypomethylated sites (52). These results altogether suggest that HBV or HCV may increase the risk of HCC development in a SALL4-dependent manner, and that SALL4 diagnostic/prognostic biomarker may perform the best in patients whose HCC is caused by hepatitis infections.

Interestingly, GPC3, SALL4, and AFP expressions are highly intertwined. There is a significant positive correlation between SALL4 expression and GPC3 expression in HCC patients, and patients with SALL4-positive HCC exhibited higher levels of AFP in serum (42, 53). In a cohort of combined HCC and cholangiocarcinoma, GPC3 immunopositivity rates were much higher in SALL4-positive samples than that of SALL4-negative samples (54). HCC patients co-expressing GPC3 and SALL4 exhibit a trend towards worse HCC-specific survival than high expression of either biomarker alone (42). Similarly, patients who coexpressed both SALL4 and AFP exhibited a significantly worse prognosis than patients who were either positive for only one biomarker or negative for both, and patients who were positive for SALL4 demonstrated a trend towards worse prognosis with increasing AFP expression levels (41). Co-expression of GPC3 and SALL4 has also been correlated with vascular invasion, poor differentiation, and higher AFP levels in HCC patients (33). Evaluating the coexpression patterns of SALL4, AFP, and GPC3 may offer opportunities for designing novel biomarker combinations and yield better insight into the patient’s disease course.

### 2.4 Oncofetal-biomarker-turned-targeted therapies

In the case of AFP, GPC3 and SALL4, the boundary between diagnostic biomarkers and therapeutic targeting are increasingly blurred, and these proteins have emerged as biomarker-guided HCC precision medicine. As tumor markers that tend to be more highly expressed in HCC cells while lowly expressed in normal hepatic cells, GPC3 and AFP can aid in the direct targeting of tumor cells by cellular therapy modalities, such as monoclonal antibodies and Car-T (55, 56). In the remaining part of this section, we briefly outline the innovative therapeutic approaches utilizing the high expression of GPC3 and AFP to identify tumor cells. Although SALL4 is also an oncofetal protein, it has distinct properties as a HCC oncogenic driver, and we will describe SALL4 targeting in the subsequent section of the paper that focuses on HCC precision medicine via targeting oncogenic pathways and mechanisms.

In HCC tumor tissues, AFP is found intracellularly and extracellularly via presentation by a major histocompatibility complex (MHC) class I molecule (57). AFP-targeting therapies have mainly utilized chimeric antigen receptor (CAR) T-cell therapy, which involves the use of genetically modified T cells engineered to recognize target antigens expressed on tumor cells and elicit an immune response (58). The ability of tumor cells to evade immune surveillance by downregulating MHC class I molecules on its surface and the intracellular localization of AFP provide considerable challenges to AFP-targeting therapies (57). While Liu et al. successfully demonstrated the antitumoral effects in preclinical HCC models by engineering AFP-CAR T cells capable of recognizing the AFP peptide presented by MHC class I molecules (59), a subsequent phase I trial (NCT03349255) with the AFP-CAR T cells was terminated (60).

Antibodies targeting GPC3, such as GC33 and HN3, have been developed and are being studied in clinical trials for the treatment of HCC (61–63). These antibodies are designed to specifically target and kill GPC3-expressing cancer cells, while sparing normal liver cells that do not express GPC3. In addition, GPC3-targeted CAR T-cell therapy is also being investigated in clinical trials for the treatment of HCC (64). These therapies have shown promising results in preclinical studies and early-phase clinical trials, highlighting the potential of GPC3 as a therapeutic target in HCC. Multiple early phase I and II clinical trials are presently studying anti GPC3 CAR T-cell therapy alone or in combination with conventional chemotherapy, or other therapeutic alternatives (65–67).

In summary, surveillance, diagnosis, progression monitoring, and prognostication of HCC can be challenging due to the lack of highly sensitive and specific biomarkers. Current diagnostic modalities centering around AFP and imaging have their respective limitations, highlighting the need for novel biomarkers and improved surveillance techniques. Incorporating composite scoring schema (e.g. GALAD and BALAD scores), and incorporating novel biomarkers such as GPC3 and SALL4, have shown promise in the diagnosis and prognostication of HCC. Excitingly, some HCC diagnostic biomarkers have found new roles in HCC therapeutic targeting, which are under active pre-clinical and clinical trial investigations.

## 3 Exploring targeted therapies in HCC: the urgent need for biomarker-guided precision medicine focusing on oncogenic pathways and transcription factors

At present, the HCC drug development landscape is dominated by immunotherapies, immunomodulators, as well as kinase inhibitors. There is a limited availability of biomarker-guided precision medicine in HCC treatment. This can be attributed to several factors. Firstly, HCC is a complex and heterogeneous disease with various underlying molecular alterations, making it challenging to identify specific biomarkers that can reliably guide targeted therapy selection. Additionally, the majority of HCC cases are diagnosed at advanced stages when curative treatments are often no longer feasible. This late-stage diagnosis limits the opportunity for biomarker identification and targeted therapy initiation. Furthermore, the liver’s unique physiology and the presence of cirrhosis in many HCC patients pose additional challenges in drug delivery and efficacy. The development of targeted therapies requires extensive research, clinical trials, and regulatory approvals, which can be time-consuming and costly. Despite these challenges, ongoing research efforts are focused on identifying novel biomarkers and therapeutic targets to advance the field of precision medicine in HCC, with the hope of improving patient outcomes in the future. In addition, it is important to emphasize that targeted therapy in HCC necessitates the availability of reliable biomarkers for patient selection and treatment monitoring. The complex nature of HCC and its molecular heterogeneity make the identification of specific biomarkers challenging. However, the integration of biomarker-guided precision medicine can enhance the efficacy and effectiveness of targeted therapies in HCC. As research progresses and novel biomarkers are identified, the development of personalized treatment approaches will become increasingly feasible, offering a promising avenue for improving patient outcomes. These insights are further corroborated by the specific mechanisms of action detailed in **Table 1**.

**Table 1:** Drugs/molecules under clinical trials targeting signaling pathways and/or transcription factors in HCC.

Drug Name	Target TFs/Molecules	Associated Signaling Pathway	Active Indications	Phase	Clinical Trial ID	Status (ongoing/completed/withdrawn)	Monotherapy/combination therapy? if combination, what are the rationales
IK-930	YAP and TEAD inhibitor	Hippo signaling	Advanced solid tumors	1	NCT05228015	Recruiting	Monotherapy
CBL-0137 (Oral and IV)	FACT complex inhibitor, TP53 gene stimulator	Apoptosis pathway	Relapsed/refractory solid tumors	1/2	NCT04870944, NCT03727789, NCT05498792	Ongoing Recruiting Recruiting	Monotherapy Monotherapy Combination with ipilimumab & nivolumab (CTLA-4 & PD-1 inhibitors) Rationale: CBL0137 has been shown to inhibit cancer stem cell growth and tumor growth, demonstrating promising results when combined with existing therapies that attack tumor cells (chemo/immunotherapy) (68)
OTX-2002	c-Myc gene inhibitor	Wnt/β-catenin, hypoxia, notch, hedgehog signaling	HCC and other solid tumors	1/2	NCT05497453	Recruiting	Combination with tyrosine kinase inhibitors & PD-1/PD-L1 inhibitor Rationale: Assessing safety of OTX-2002 in combination with standard HCC treatments; c-Myc inhibition restricts tumor growth and may augment other therapies that also control tumor expansion
YIV-906	Beta-glucuronidase, cytochrome P450 3A4, metalloprotease-2 and NF-KB inhibitor, NK-1 receptor and opioid receptor delta antagonist	Apoptosis pathway	HCC, pancreatic tumor	2	NCT04000737	Recruiting	Combination with Sorafenib Rationale: Clinical/preclinical research suggests that YIV-906 can increase the antitumor activity of sorafenib (69)
TLC-388	HIF1α and topoisomerase I inhibitor	Hypoxia signaling	Advanced HCC, differentiated neuroendocrine carcinomas, advanced/metastatic renal cell carcinoma	1/2	NCT00747474, NCT02457273, NCT01831973	Completed, stable disease in 51% of patients (n = 41) (70) Completed, stable disease in 15% of patients (n = 20), median PFS 1.8 months & median OS 4.3 months (71) Completed	Monotherapy Monotherapy Monotherapy
BN-Brachyury	Brachyury protein modulator	Brachyury signaling	Advanced solid tumors	1/2	NCT03493945, NCT05445882	Recruiting Not yet recruiting	Combination with N-803 (immunotherapy) Rationale: T-cells were shown to be activated against brachyury in a phase I clinical trial. N-803 stimulates cytotoxic T cells and NK cells, and it may augment antitumor effects of BN-Brachyury (72–74)
NKT-2152	HIF2α inhibitor	Hypoxia signaling	HCC, metastatic renal cell carcinoma	1/2	NCT05119335	Recruiting	Monotherapy
DSP-7888	WT1	Wnt/β-catenin signaling	Advanced solid tumors	1/2	NCT04747002, NCT02498665	Recruiting Completed	Monotherapy Monotherapy

HCC, hepatocellular carcinoma; NSCLC, non-small cell lung cancer; YAP, yesassociated protein; TEAD, transcriptional enhanced associate domain; EGFR, epidermal growth factor receptor NF-KB, nuclear factor kappa B; NK, neurokinin; TP53, tumor protein p53; HIF, hypoxia-inducible factor; WT1, Wilms tumor 1-associating protein; PFS, progression-free survival; OS, overall survival.

**Figure 2:**
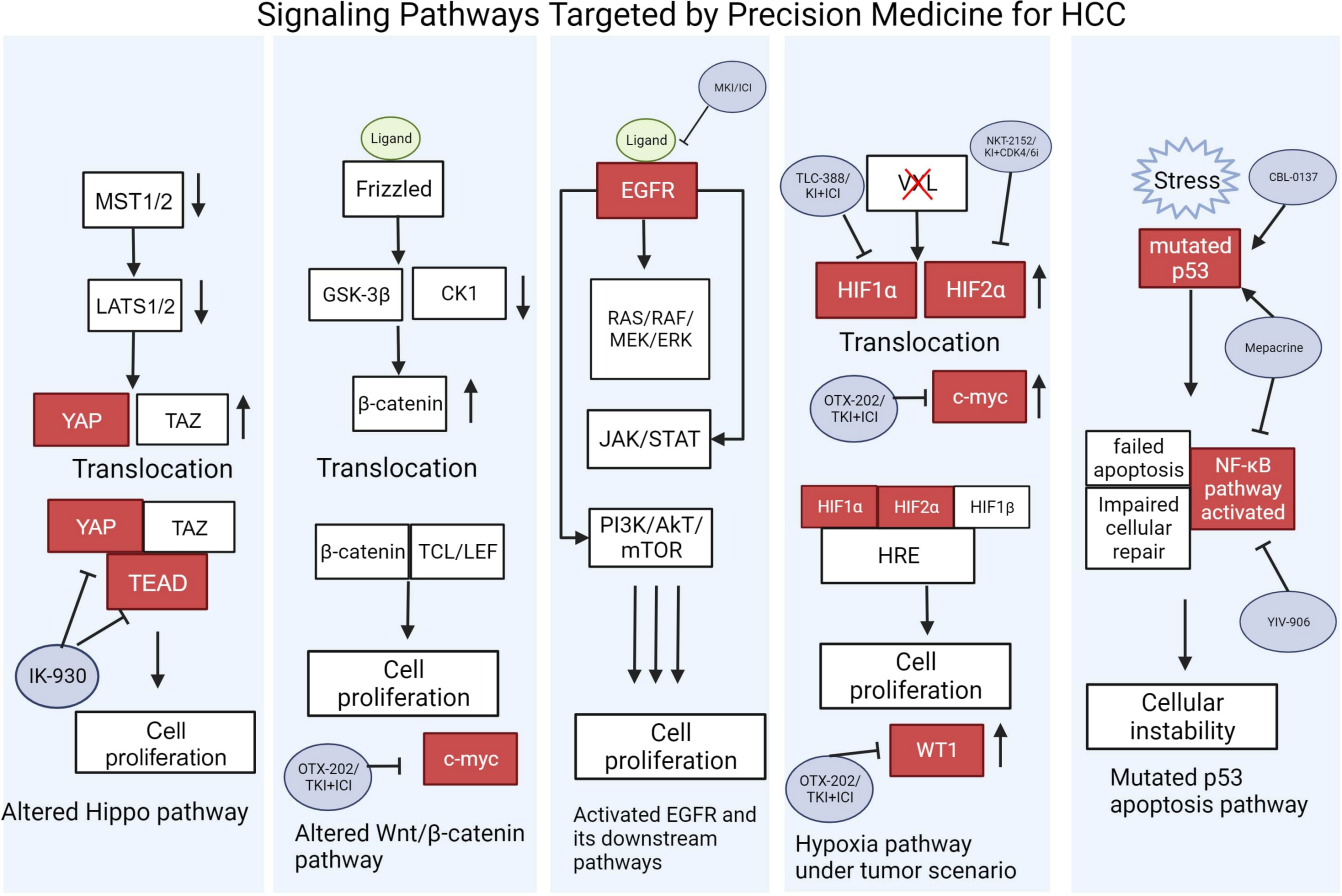
Signaling Pathways Targeted by Precision Medicine for HCC. Pathways are shown in their altered state caused by ligand signaling, stress, or gene mutations that lead to eventual cell proliferation. Gene activation and repression are illustrated with up or down arrows. TFs targeted by potential drugs under clinical trials are shown in red. Refer to **Table 1** for the full descriptions of the drugs used to target these oncoproteins. This figure was created with BioRender.com.

**Figure 3:**
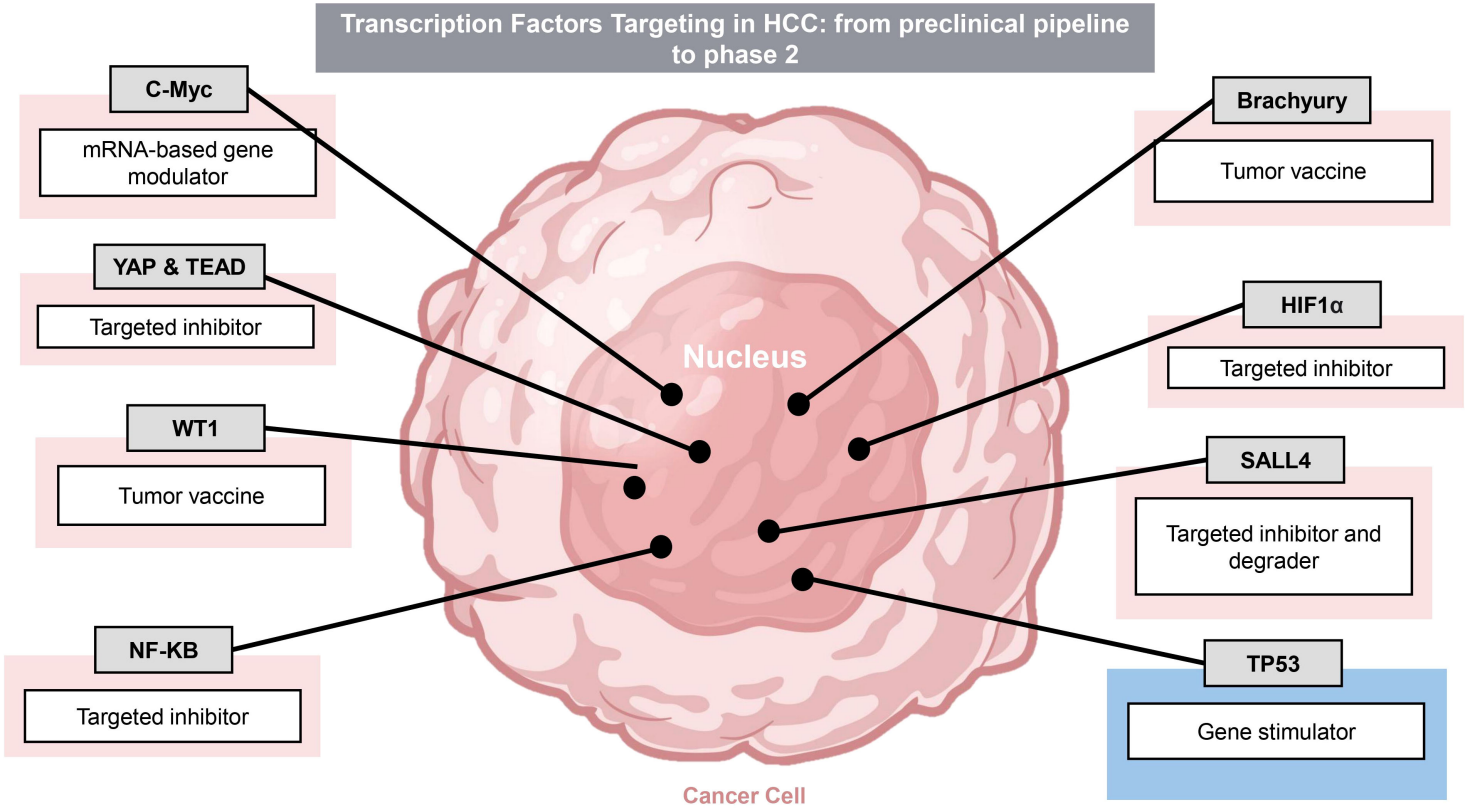
Transcription Factor targets in HCC. There are multiple drug candidates targeting transcription factor oncoproteins and tumor suppressor under pre-clinical and clinical trial development. Transcription factors and targeting modalities are summarized. Pink shading indicates oncoproteins whereas blue indicates tumor suppressors.

There is substantial effort today in both preclinical and clinical trials assessing the efficacy of interventions targeting the key oncogenic pathways in HCC. Here, we review the pathophysiology of several well-characterized, dysregulated pathways, with a focus on oncogenic TFs (**Figure 2**). TFs, critical for cancer development and survival, historically have been viewed to be “undruggable”. However, recent breakthroughs and successes in targeting TFs are among the most exciting new frontiers for development of novel cancer drugs. There are a total of 9 TF-targeting HCC drugs in phase 1-3 development, spanning 8 different targets (**Figure 3**, **Table 1**).

### 3.1 Hippo signaling

The Hippo pathway consists of a kinase cascade involving the mammalian Ste20-like kinases 1/2 (MST1/2; homologs of Drosophila Hippo), which controls cell growth by phosphorylating the large tumor suppressor 1/2 (LATS1/2; homologs of Drosophila Warts). The cascade leads to the inhibition of two transcriptional coactivators: the Yes-associated protein (YAP) and transcriptional co-activator with PDZ-binding motif (TAZ) (75). By interacting with α-catenin and the 14-3-3 protein, LATS1/2 subsequently induces the phosphorylation of YAP and TAZ, resulting in their degradation by Casein kinase 1δ/ϵ and SCF E3 ubiquitin ligase or cytoplasmic sequestration by the 14-3-3 protein (75, 76). In the absence of Hippo signaling, YAP and TAZ translocate into the nucleus to interact with the transcriptional enhanced associate domain (TEAD) family. The activation of TEAD induces the expression of genes related to cell growth, survival, migration, anti-apoptosis and epithelial-mesenchymal transition (EMT) (76, 77).

In the context of HCC, the overexpression of YAP and TAZ was significantly associated with the increased proliferative activity of tumor cells. In HCC cells and human HCC samples, TAZ was expressed at higher levels than YAP with TAZ mRNA levels having a positive association with poor outcomes. While TAZ knockdown can attenuate cell growth by inhibiting the PI3K/AkT/mTOR signaling pathway, it additionally leads to a compensatory YAP upregulation and expression of CD90, a HCC-specific tumor stem cell marker. Moreover, continuous 5-fluorouracil treatment was shown to reduce TAZ expression and induce YAP and CD90 mRNA expression to confer HCC cells chemoresistance. These findings suggest that targeting both YAP and TAZ or its downstream target TEAD may be crucial to suppress HCC oncogenesis mediated by the Hippo pathway (78).

IK-930 is a small molecule inhibitor of TEAD currently in phase 1 clinical trial to evaluate its safety, efficacy, and tolerability in patients with advanced solid tumors (79). Besides binding to TEAD, IK-930 promotes interactions between TEAD and vestigial like family member 4 (VGLL4), a negative regulator of the Hippo signaling pathway, to inhibit the transcriptional activity of TEAD (80, 81). IK-930 demonstrated selectivity for Hippo-mutated cells and potent TEAD inhibition *in vitro* as well as a robust antitumor activity in preclinical models of mesothelioma and head and neck squamous cell carcinoma with mutations in the Hippo pathway (82). Throughout the phases of its clinical trial, it remains to be seen whether IK-930 can serve as a powerful addition to the arsenal of treatments for Hippo-mutated HCCs.

### 3.2 Wnt/β-catenin signaling

In normal adult tissues, the wnt/β-catenin signaling pathway is inactive and regulated by wnt antagonists and the β-catenin destruction complex. The binding of wnt ligands to one of the frizzled receptors and one of the low-density lipoprotein receptor-related protein (LRP) 5/6 co-receptors activates the pathway, recruiting Disheveled (Dsh) and AXIN scaffolding proteins to the membrane (83, 84). The subsequent degradation of AXIN leads to the stabilization of β-catenin by disassembling its degradation complex, enabling β-catenin accumulation in the cytoplasm and its translocation into the nucleus (85). Inside the nucleus, β-catenin binds to TFs belonging to the T-cell factor/lymphoid enhancer-binding factor to induce the expression of downstream targets controlling liver cell proliferation, differentiation, and development (83, 84).

40-70% of HCCs exhibit β-catenin accumulation in the nucleus due to mutations in the β-catenin gene allowing β-catenin to avoid degradation (84). Such mutations have been found more frequently in cells with loss of function mutations in the Wilms tumor 1 (WT1) gene, an antagonist and negative regulator of the wnt/β-catenin pathway (86). The binding partner to the WT1 protein, Wilms’ tumor 1-associating protein (WTAP), has been suggested to have an oncogenic role in many tumor types. In HCC, the overexpression of WTAP has been correlated to poor outcomes by the post-transcriptional suppression of ETS proto-oncogene 1 (ETS1), a tumor suppressor (87). In the context of colon cancer, targeting and degrading Wilms’ tumor 1-associating protein (WTAP) with carbonic anhydrase IV successfully restored free WT1 proteins capable of inhibiting the Wnt/β-catenin pathway, demonstrating the potential benefits of WTAP-targeting therapies (88). In addition, downstream targets of the wnt/β-catenin signaling pathway responsible for HCC proliferation and metastasis, such as c-Myc and leucine-rich repeat-containing G (LGR5), respectively, may be considered as potential targets for therapy (84).

### 3.3 Epidermal growth factor receptor signaling

EGFR is a transmembrane tyrosine kinase receptor that binds to ligands such as the epidermal growth factor (EGF) and transforming growth factor α (TGF-α). Upon its activation, it induces downstream pathways involved in cell proliferation and differentiation, such as the Ras-Raf-MEK-ERK pathway and the JAK/STAT pathway. In addition, it can activate the PI3K/Akt/mTOR pathway responsible for controlling cell survival (89). With regards to HCC, EGFR overexpression is observed in 68% of HCCs and is positively correlated to poor outcomes (90, 91). Increased EGFR expression has been shown to enhance the microvessel density of tumors and further promote their proliferation (92). Similarly, the overexpression of EGFR ligands such as EGF and TGF-α was confirmed by multiple studies using human HCC specimens (92–94). The expression of betacellulin, which belongs to the EGF family, in HCC cells has been shown to have a significant positive correlation to EGFR expression by tumor endothelial cells, suggesting a potential paracrine signaling pathway to induce tumor angiogenesis (92).

Recently, Jin et al. reported that EGFR depletion increased the antitumoral activity of lenvatinib towards HCC cells using a CRISPR/Cas9-based synthetic lethality screening. The combination of MKI, lenvatinib and the EGFR inhibitor gefitinib exhibited robust anti-proliferative effects in liver cancer cell lines as well as in murine preclinical models xenografted with human liver tumors. The two treatments additionally increased the infiltration of natural killer (NK) cells and CD8+ cytotoxic T cells into the tumor microenvironment, suggesting that ICI in combination with lenvatinib and gefitinib may lead to a more potent clinical response (95). In a phase I clinical study on 12 HCC patients, 4 patients demonstrated a partial response, 4 exhibited stable disease states, and 4 had disease progression after 4 to 8 weeks of the combination treatment (96).

### 3.4 Hypoxia signaling

Solid tumors in HCC generate hypoxic conditions within the tumor microenvironment due to poor vascularization and increased metabolism. The hypoxic environment prevents the degradation of hypoxia inducible factor (HIF) α subunits by reducing the activity of prolyl hydroxylase domain-containing proteins (97). Increased expressions of HIF1α and HIF2α have been observed in multiple tumor types; however, the TFs have differing roles across distinct cell types. For example, the loss of HIF2α in a KRAS-driven lung tumor model surprisingly increased tumor progression, while HIF2α deficiency in mouse vascular endothelial cells reduced tumor expansion. HIF1α and HIF2α additionally exhibit differential activities, sometimes demonstrating opposite effects with regards to cancer progression (98).

In normal cells, hypoxia arrests cell growth through HIF1α, which induces c-Myc degradation to decrease anabolic metabolism and protein synthesis. In contrast, transformed cells exclusively expressing HIF2α enhance c-Myc activity under hypoxic conditions, promoting cell growth and proliferation (99). Elevated c-Myc expression is additionally capable of overriding HIF1α-mediated inhibition, where HIF1α and c-Myc together increase cell proliferation (98, 100). Moreover, c-Myc can post-transcriptionally promote HIF1α expression and further promote tumor growth (101). In HCC, the overexpression of both HIF1α and HIF2α have been observed and associated with poor prognosis (97). A study by Mu et al. demonstrated that HIF2α, but not HIF1α, was positively correlated to both c-Myc expression and the Union for International Cancer Control tumor stages in human HCC tissues. Knockdown of HIF2α successfully inhibited c-Myc expression and restricted HCC growth, elucidating it as a potential target for therapy (102).

TLC388 is a combination of HIF1α and nuclear DNA topoisomerase 1 (TOP1) inhibitors that underwent a phase I clinical trial on 54 patients with advanced solid tumors from 2008 to 2011 (103). In the study, 13 patients did not exhibit any clinical response, while among 41 evaluable patients, 21 exhibited stable disease. Overall, TLC388 was well-tolerated and demonstrated an ability to prolong a stable disease state in multiple tumor types (70). While the study did not progress onto later phases, it is much desired to evaluate the effects of TLC388 in combination with other therapeutic agents, such as EGFR inhibitors or ICI, for HCC treatment. Similarly, NKT2152 is an orally administered HIF2α inhibitor demonstrating potent reduction in tumor growth in murine preclinical models of clear cell renal cell carcinoma (ccRCC) and other solid tumors including HCC. A combination of NKT2152 and VEGFR or cyclin-dependent kinase (CDK) 4/6 inhibitor increased antitumoral effects (104). While NKT2152 is currently undergoing a phase II clinical trial in patients with ccRCC, it remains to be seen whether combination of NKT2152 and kinase inhibitors can elicit robust clinical response in HCC patients (105).

Targeting a downstream target of HIF, OTX-2002 is a mRNA therapeutic that pre-transcriptionally modulates c-Myc gene expression. It demonstrated an ability to downregulate c-Myc expression in HCC cells *in vitro* and elicited potent antitumoral activities in murine HCC xenograft models (106). OTX-2002 is currently undergoing a phase I/II clinical trial in patients with HCC or other solid tumors with c-Myc involvement to determine its antitumoral activity alone or in combination with other treatments such as tyrosine kinase inhibitors (TKI) or ICI (107, 108).

### 3.5 TP53 dependent pathways

In HCC, TP53, a critical tumor suppressor gene, plays a multifaceted role. TP53’s primary function is to monitor and maintain genomic stability by preventing the growth of cells with damaged DNA. In HCC, TP53 mutations or dysregulation are frequent, contributing to the initiation and progression of liver cancer (109). As a transcription factor, TP53 regulates the expression of target genes that can promote cell cycle arrest, apoptosis, DNA repair, among other oncogenic processes (110). This loss of TP53 function in HCC not only promotes tumor growth but also makes cancer cells more resistant to therapy, rendering it a pivotal factor in the development and treatment of HCC (109). Multiple efforts have been devoted to directly or indirectly target the TP53 dependent pathways in HCC, to restore or potentiate the tumor suppressor function of TP53 (110). Candidates in the clinical trial pipeline are summarized in **Table 1**.

### 3.6 WT1

Despite its tumor-suppressive activities, elevated WT1 expression has been discovered in tumors derived from epithelial, mesenchymal, hematopoietic, and neuronal tissues, suggesting its paradoxical oncogenic function (111). In fact, in hypoxic conditions, HIF can activate WT1 in vascular cells to promote angiogenesis at the tumor site. Silencing WT1 expression decreased the expression of the TF ETS-1 in addition to reducing endothelial cell migration, proliferation, and vascular formation (112). In HCC, the downregulation of WT1 by the antisense of WT1, a long non-coding RNA, was shown to promote apoptosis and restored sensitivity to chemotherapy (113). Thus, targeting WT1 can serve as a promising approach to restrict tumor growth by inhibiting angiogenesis and chemoresistance. DSP-7888 (Ombipepimut-S) is a cancer vaccine composed of synthetic epitopes nelatimotide and adegramotide. Nelatimotide contains two CD8+ cytotoxic T cell epitopes of WT1, while adegramotide contains a targeting sequence for the endogenous WT1 protein. By stimulating WT1-specific cytotoxic and helper T cells, DSP-7888 in combination with anti-programmed cell death protein 1 (PD-1) demonstrated its ability to elicit strong antitumoral activity toward cancer cells expressing high levels of WT1 during *in vitro* and *in vivo* preclinical trials. For a robust immune response, a combination therapy with ICI may be the key to prevent the reduced activity of immune cells at the tumor microenvironment (114). In combination with ICI, DSP-7888 has completed multiple phase I clinical trials, including those on patients with advanced solid tumors. Currently, DSP-7888 is undergoing a phase II clinical trial for acute myeloid leukemia patients with complete remission (115).With regards to targeting the wnt/β-catenin pathway in HCC, a multidimensional approach may be required due to its complexity. It is implicated that β-catenin may have multiple roles in HCC progression than its transcriptional regulation; in fact, β-catenin nuclear accumulation was found to be restricted to late-stage HCC. In early stage HCC cells, increased expression of E-cadherin leads to β-catenin recruitment to the plasma membrane by the AJ complex, a complex previously known to regulate intercellular interactions and maintain the F-actin cytoskeleton. At the membrane, β-catenin and the AJ complex promotes EGFR stabilization and signaling, which allows tumor survival (116). Moreover, murine models have showcased an increase in macrophages and a decrease in CD4+ helper T cells as the mice progressed from steatosis to cancer. The isolated macrophages exhibited high expression of wnt ligands to activate the wnt/β-catenin pathway to induce tumorigenesis during steatosis (117).

### 3.7 Brachyury

Brachyury is a T-box TF involved during vertebrate development and frequently dysregulated in neoplastic diseases such as colorectal cancer (118, 119). In a study on oral squamous cell carcinoma cells, Brachyury expression was found to be correlated with EMT and lymph node metastasis, and increased Brachyury expression was discovered in human HCC cell lines (120, 121). Brachyury moreover increases the invasiveness and metastatic potential of HCC cells and promotes EMT in HCC cells through the Akt/Snail pathway (121). Current therapies targeting the Brachyury TF include the BN-brachyury cancer vaccine, which employs a modified vaccinia Ankara (MVA) virus to elicit a robust immune response toward solid tumor cells overexpressing the Brachyury protein (72). Silencing Brachyury expression has demonstrated an ability to reduce the invasiveness of non-small cell lung carcinoma cells *in vitro* and *in vivo* using xenograft mice (122). A phase I trial of the MVA-BN-brachyury cancer vaccine on 13 patients with advanced cancers yielded one patient with a partial response, 4 with stable disease states, and 8 with progressive disease. While meaningful T cell activity was observed in these patients, it remains to be seen whether the potency of the response increases in combination with ICI or if prior immunotherapy treatment is necessary for a response (72). Currently, the MVA-BN-brachyury cancer vaccine in combination with anti-PD-L1 ICI is undergoing a phase I/II study on patients with advanced solid tumors (123).

### 3.8 SALL4

Another promising TF target is SALL4, a TF involved in cell renewal and growth during development (124). While SALL4 is silenced in most adult tissues, its reactivation can lead to malignant neoplasms (125). SALL4 acts downstream of multiple signal transduction pathways, including JAK/STAT and Wnt/β-catenin (126, 127). Upon its activation, SALL4 acts on multiple signaling pathways involved in cell growth, such as PI3K/Akt/mTOR, lysine demethylase 3A (KDM3A), Forkhead-Box (FOX), and c-Myc genes, the latter which it binds directly to the promoter to elevate its expression (128–130). SALL4 therapeutic targeting in HCC is currently in the pre-clinical development stage. Restricting the interaction between SALL4 and Nucleosome Remodeling and Deacetylase complex (NuRD), a corepressor, with a peptide competitive inhibitor reduced the viability of SALL4-overexpressing HCC cells *in vitro* and *in vivo* (40, 131). A number of other SALL4-targeting modalities, such as small molecule degraders, are also under active pre-clinical development. SALL4-based precision medicine relies on companion diagnostics to successfully select HCC patients who express high levels of SALL4; efforts are under way to develop an ultrasensitive peripheral blood protein assay to be used in conjunction with SALL4-targeting therapies.

## 4 Currently approved and ongoing clinical trials of immunotherapy and targeted therapy combinations

The utilization of combination therapies in HCC has gained significant traction in recent years. Until 2017, the utilization of antiangiogenic MKIs was a common approach in systemic therapy for advanced-stage HCC patients. These MKIs target angiogenesis pathways and aim to disrupt tumor vasculature, thereby inhibiting tumor growth and progression. However, the introduction of immunotherapy with ICI, such as PD1/PDL1 inhibitors, has provided an additional therapeutic avenue. ICI stimulates effective anti-tumor activity by unleashing the immune system’s ability to recognize and attack cancer cells. The ICIs have demonstrated efficacy in the clinical management of HCC (132). The simultaneous targeting of VEGF-mediated angiogenesis and immune checkpoints hold promise for improving treatment outcomes in HCC (133). The success of these earlier generations of combination therapies are paving ways for the ongoing development of novel combination approaches. In fact, many of the TF/signaling pathway-targeting modalities (e.g. cMYC targeting) and biomarker guided monoclonal antibody/cell therapy approaches (e.g. anti-GPC3, or GPC3 CAR-T) center around combination therapy in the pre-clinical and clinical development stages, mostly in combination with existing first line therapies.

In this section, we explore some well-known examples of combination therapies, to highlight a few core themes and considerations - 1) the importance of mechanism-driven rationale design, 2) consideration of drug sequencing, 3) need for robust companion biomarkers, and 4) optimization safety profile - to guide development of future combination therapies in light of the many exciting, emerging HCC novel targets. This section will focus on combination medical therapies alone, but it is important to note that combining medical therapies (e.g. ICIs) with locoregional therapies, such as transarterial chemoembolization (TACE) or ablation, has been found to be well-tolerated and potentially efficacious in certain subsets of HCC patients (134) (135). The discussion of combination surgical and medical therapies are beyond the scope of this review.

### 4.1 In-depth mechanistic investigation inspired rational design of dual VEGF-targeting and ICI

Animal models have been instrumental in studying the effects of VEGF/VEGFR pathway blockade in combination with PD1 inhibitors leading to its clinical development, highlighting the importance of in-depth mechanistic investigation in guiding the rationale design of novel combination approaches (136). Studies in animal models have provided insights into the specific changes that occur in the TME following the dual blockade of VEGFR and PD1. These changes include a reduction in tumor vasculature, a decrease in myeloid-derived suppressor cells (MDSCs), an increase in M1-like tumor-associated macrophages (TAMs) and infiltrating CD8+ cells, and a decrease in M2-like TAM levels. These findings highlight the complex interactions between immune cells, tumor cells, and the vasculature in the context of combination therapy (136).

Backed by strong pre-clinical rationale, the combination of the anti-PDL1 antibody atezolizumab and the VEGF-neutralizing antibody bevacizumab has emerged as a first-line therapy for HCC. Its approval by the FDA in 2020 marked a significant milestone in HCC treatment. In the clinical trial setting, it has demonstrated superior efficacy over sorafenib in the frontline setting for advanced HCC (132).

Other HCC pathogenic drivers/pathways have been shown to interact with the immune checkpoint pathway, and a deeper mechanistic understanding of their interplay could guide rational design of novel combination therapies. For example, recent studies have implicated SALL4 in the regulation of PD-1/PD-L1-mediated T cell exhaustion in HCC (137, 138). Another study found that SALL4-mediated upregulation of exosomal miR-146a-5p drives T-cell exhaustion by M2 tumor-associated macrophages in HCC (30). We anticipate more innovative therapies in the near future that harness synergistic anti-tumor mechanisms between HCC oncogenic pathways and ICIs.

### 4.2 Treatment sequence in the setting of combination therapies

Sorafenib, a MKI targeting both VEGFRs and PDGFR-b, was the first targeted therapy approved for advanced HCC. Other MKIs, including regorafenib and lenvatinib, have also shown potential in improving survival outcomes for HCC patients. Combining MKIs and ICIs recently became a popular combination therapy strategy in HCC, but clinicians often face uncertainties in using this combination. Rational design involves understanding of how therapy efficacy can be affected by sequencing, among others.

Several clinical trials have investigated or are investigating this combination using different treatment sequencing. A propensity score-matching study comparing simultaneous administration of anti-PD-1 combined with sorafenib versus anti-PD-1 alone in advanced HCC showed that the combination therapy had better efficacy and survival benefits (139). The combination therapy exhibited a higher complete response rate, overall response rate, disease control rate, and achieved more tumor shrinkage compared to anti-PD-1 alone. Additionally, the combination therapy demonstrated longer progression-free survival and a decreasing risk of disease progression and death. There were no statistically significant differences in grade 3/4 toxicities between the two treatment options, except for one case of ‘sick sinus syndrome’ that developed with combination therapy (14). Sequential therapies have also been explored with anti-PD1 antibodies, such as nivolumab and pembrolizumab, being used after MKI in the treatment of HCC. This is still being explored in the clinical trial setting (140), but it is being used empirically in the clinical setting (141). Rational design of these two combinations, whether administered sequentially or simultaneously, is an important consideration for optimizing treatment outcomes.

### 4.3 Combination therapy approaches involving multiple ICIs

Simultaneous administration of multiple sub-classes of ICIs, such as the combination of CTLA4 and PD-L1 blockade, have been explored for HCC treatment. Preclinical studies and clinical trials have demonstrated synergistic effects when both pathways are targeted simultaneously, leading to enhanced anti-tumor immune responses (142). The rational design of this combination involves understanding the complex interplay between CTLA4 and PD-L1 signaling. The use of multiple ICIs are limited due to various reasons, but notably the current lack of robust biomarkers for patient selection. Tumor mutation burden (TMB) and microsatellite instability (MSI) have been used as a biomarker for predicting ICI therapy efficacy, but no single biomarker has been established as a reliable predictor of response to immunotherapy in HCC (143).

Additional exploratory biomarkers in tissue analysis include tumor gene expression profiling (GEP), multiplex immunohistochemistry (IHC), immunofluorescence (IF), tumor infiltrating lymphocytes (TILs), immunoscore, T cell receptor (TCR) diversity, and microbiome (144, 145) New assays such as macroH2A1 staining and stemness-associated genes are being explored to identify patients likely to respond to ICIs (146, 147). Many ongoing clinical trials involving ICIs also have concurrent exploratory companion biomarker designs, so it is likely that in the near future, more biomarkers will be validated to help strategy patients who may benefit from different types of immune-check point inhibition, or their combinations.

### 4.4 Rational design of combination therapies: patient safety considerations

Rationale design of therapy combination should also take into account patient-specific factors, contraindications, and potential risks associated with each therapy alone or in combination. For example, combining ICIs and TKIs can lead to more severe toxicity. Skin lesions, diarrhea, and hepatitis are some examples of adverse events associated with this combination that can be more severe than single therapy alone (148). Lethal adverse events have been reported with the combination of pembrolizumab and lenvatinib in HCC patients. The potential for severe adverse events highlights the need for careful risk-benefit assessment and close monitoring during treatment. Patient-reported outcomes and quality-of-life assessments should be considered alongside efficacy data when designing and evaluating the overall impact of combination therapies (149).

The VEGF inhibitor Bevacizumab has been associated with an increased risk of gastrointestinal hemorrhage or perforation in patients with advanced cancer. Notably, there was no difference in the occurrence of these adverse events between cirrhosis patients and cancer patients, indicating that the risk is not limited to specific subgroups (150). Considering the pathophysiology of portal hypertension, VEGF inhibition may not aggravate portal pressure and should be evaluated in the context of individual patient characteristics. Comparing different combination approaches, the combination of atezolizumab and bevacizumab has shown better tolerability compared to the combination of ICIs and TKIs. This finding suggests that certain combinations may have distinct toxicity profiles, and rational design should consider the potential impact on patients’ well-being (139).

Toxicities associated with combination therapies may not be entirely discouraging. In fact, they might indicate an increased chance of treatment benefit. For instance, skin toxicity associated with sorafenib has been associated with improved outcomes in HCC patients (151). This suggests that a comprehensive understanding of toxicity patterns and their relationship to treatment response is important for rational decision-making. In a single-center cohort study of HCC patients, the presence of immune-mediated adverse events (IMAEs) was independently associated with improved median progression-free survival (PFS) and overall survival. This finding suggests that the occurrence of IMAEs may serve as a positive prognostic factor and warrants further investigation (152).

## 5 Conclusion

The field of HCC treatment is rapidly evolving, with a focus on combination therapies that target multiple pathways and mechanisms. The rational design of these combination therapies involves understanding the complex interactions between different agents, considering their distinct toxicity profiles, and identifying patient-specific factors to guide treatment decisions. Targeted therapies, such as those targeting specific signaling pathways and TF oncogenes, can be developed based on a better understanding of the molecular and genetic alterations that drive HCC progression and disease heterogeneity.

The future scope of HCC research and treatment lies in the development of more effective targeted therapies and personalized treatment strategies, preferably guided by biomarkers. The ongoing effort to identify and validate novel biomarkers for HCC diagnosis, prognosis, and treatment response will allow for the selection of appropriate therapies for individual patients as the treatment options expand. Overall, The integration of biomarker-guided precision medicine and the exploration of targeted therapies and combination approaches are expected to shape the future landscape of HCC treatment and pave the way for more personalized and effective treatment strategies.

## Author contributions

JL: Conceptualization, Writing – original draft, Writing – review & editing. KP: Writing – original draft. ZS: Writing – review & editing. HL: Writing – original draft, Validation. PG: Writing – original draft, Writing – review & editing. MP: Writing – original draft. LC: Conceptualization, Funding acquisition, Writing – original draft, Writing – review & editing.
